# Advancing the activity cliff concept

**DOI:** 10.12688/f1000research.2-199.v1

**Published:** 2013-09-30

**Authors:** Ye Hu, Dagmar Stumpfe, Jürgen Bajorath

**Affiliations:** 1 Department of Life Science Informatics, B-IT, LIMES Program Unit Chemical Biology and Medicinal Chemistry, Rheinische Friedrich-Wilhelms-Universität, Bonn, D-53113, Germany

## Abstract

The activity cliff concept has experienced increasing interest in medicinal chemistry and chemoinformatics. Activity cliffs have originally been defined as pairs of structurally similar compounds that are active against the same target but have a large difference in potency. Activity cliffs are relevant for structure-activity relationship (SAR) analysis and compound optimization because small chemical modifications can be deduced from cliffs that result in large-magnitude changes in potency. In addition to studying activity cliffs on the basis of individual compounds series, they can be systematically identified through mining of compound activity data. This commentary aims to provide a concise yet detailed picture of our current understanding of activity cliffs. It is also meant to introduce the further refined activity cliff concept to a general audience in drug development.

## Introduction

Activity cliffs have been discussed in the medicinal and computational chemistry literature since the early 1990s ^1– 4^. In the practice of medicinal chemistry, structurally similar compounds with large potency differences are often encountered, mostly during the chemical optimization of individual compound series. Moreover, activity cliffs have also been systematically identified by computational compound data mining ^3, 4^, which has sparked further interest in the activity cliff concept. Hence, in addition to the study of activity cliffs on a case-by-case basis in medicinal chemistry, a large knowledge base of activity cliff information is emerging. In addition, in recent years, the activity landscape concept has also become popular ^5^. Activity landscapes are generally defined as graphical representations that integrate similarity and potency relationships of compounds sharing the same biological activity ^5^, and activity cliffs are their most prominent features ^3, 5^. As compound data sets rapidly grow in size, activity landscape representations are increasingly used as tools for SAR visualization ^6^, which further emphasizes the notion of activity cliffs. Hence, the activity landscape and cliff concepts go hand in hand.

Two recent perspective articles have provided detailed accounts of activity cliff research and new developments ^3, 4^. For an in-depth review of the activity cliff research area, the interested reader is referred to these publications and the references therein. This commentary does not aim to present a full account of activity cliffs and their utility in drug discovery. Rather, it aims to distill out the information that is most relevant to provide a differentiated and critical, yet easy-to-understand view of activity cliffs. In addition, some new findings are reported concerning the target distribution of activity cliffs and coordination of cliffs, which further complement the picture. As mentioned above, computational approaches have substantially influenced our current understanding of activity cliffs. Since we strive for a widely accessible presentation of the activity cliff concept, the discussion of computational details is kept herein to an essential minimum. Furthermore, given that a number of recent activity cliff investigations have originated from our laboratory, some of the views and recommendations presented herein are at least partly subjective. However, it is hoped that they might, nevertheless, stimulate further exploration and discussion of the activity cliff concept. Several recommendations made should also aid in practical applications.

## Definition-related key aspects

An activity cliff has originally been defined as a *pair of structurally similar compounds with a large difference in potency* ^2, 3^. This general definition has four key aspects, which require further consideration and specification:

*(i)* Only a pair of compounds is considered.

*(ii)* Both compounds are active (against the same target).

*(iii)* A *structural similarity criterion* must be specified (i.e., how is *similarity* assessed and how *similar* must compounds be?).

*(iv)* A *potency difference criterion* must be established (i.e., when is a *potency difference* considered to be *large*?).

In the following, these key points will be further evaluated (in reverse order).

### Potency difference criterion

To clearly establish the potency difference criterion, it must not only be decided how large a potency difference between two compounds should be but also considered which type of potency measurements should be utilized. We emphasize that activity cliff information is only useful if the description of cliffs is accurate and interpretable ( *vide infra*). This also relates to potency comparisons. Different types of potency measurements should not be combined, e.g., assay-dependent IC _50_ measurements and (in theory) assay-independent equilibrium constants (K _i_ values) should be separately considered. Moreover, the use of approximate potency annotations (such as "% inhibition") should be avoided to ensure that SAR information encoded by activity cliffs is accurate. As our understanding of activity cliffs has evolved over the years, we have become increasingly conservative in the assessment of cliffs ("conservatism" will indeed be a recurrent theme in our discussion). Therefore, we generally prefer K _i_ values ( *vide infra*), which are in principle the most accurate measurements.

Concerning the magnitude of potency differences, there is no generally applicable rule for the definition of activity cliffs. We have found that statistical significance assessment typically yields data set-dependent results. On the basis of large-scale SAR exploration of many different data sets, we have also concluded that the presence of an at least 100-fold difference in potency as a cliff criterion generally leads to the identification of "significant" activity cliffs in compound data sets from which SAR information can often be deduced. Clearly, this represents a heuristic and not a rule written in stone.

### Similarity criterion

Without doubt, the assessment of compound similarity is the most difficult task for activity cliff definition and analysis, for several reasons. The quantification of compound similarity is strongly dependent on chosen molecular representations (descriptors). In addition, there are no generally accepted similarity measures. For activity cliff definition, the calculation of Tanimoto similarity ^7^ on the basis of different fingerprint representations ^7– 9^ has thus far been most popular ^3^. Fingerprints are generally defined as bit representations of molecular structure and/or properties. As such, they are fairly abstract descriptions of compounds. Two fingerprints of different design that have often been used for the description of activity cliffs are the "molecular access system (MACCS) structural keys" ( http://accelrys.com) ^8^, one of the "classical" fingerprints, and the "extended connectivity fingerprint with bond diameter 4 (ECFP4)" ^9^, a more recent design. MACCS consists of a set of 166 defined structural fragments whose presence or absence in a compound is monitored and ECFP4 is a topological fingerprint that generates varying numbers of atom environments for test compounds. These fingerprints are calculated from molecular graphs and are thus 2D representations. Furthermore, for the purpose of our discussion, it is sufficient to appreciate that the Tanimoto coefficient (Tc) is a similarity measure that ranges from 0 to 1 and quantifies fingerprint overlap as a measure of molecular similarity (i.e., a Tc value of 0 is produced by fingerprints that share no features and a value of 1 by identical fingerprints). A MACCS Tc value of 0.85 (corresponding to Tanimoto similarity of 85%) has often been applied as a similarity criterion for activity cliff formation ^3^. This value approx. corresponds to an ECFP4 Tc value of 0.56 because the same percentage of compound pairs reaches or exceeds these MACCS- and ECFP4-dependent values in systematic compound comparisons ^4^. Because computational similarity methods are strongly representation- and compound class-dependent, the assessment of activity cliffs suffers from the same dependencies. Consequently, activity cliff distributions often vary significantly dependent on the representations and similarity measures used ^10, 11^. Furthermore, calculated fingerprint Tc values are often difficult to interpret from a medicinal chemistry point of view ^3– 5^, which further complicates matters.

**Figure 1.**
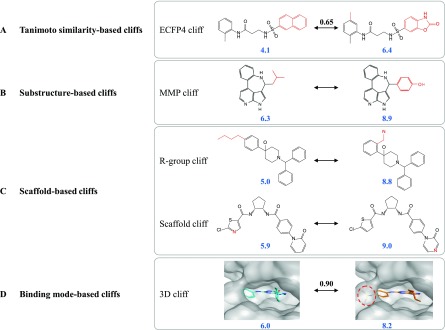
Categorization of activity cliffs. In ( **A**)–( **D**), four categories of activity cliffs are shown. Structural differences between activity cliff compounds are highlighted (red). For ECFP4 ( **A**) and 3D cliffs ( **D**), calculated similarity values are reported, i.e., 0.65 and 0.90 refer to the value of the Tanimoto and property density function similarity coefficient ^17^, respectively (both of which range from 0 to 1). Compound potency (pK _i_) values are given in blue.

In light of these difficulties, attempts have been made to replace calculated similarity values for activity cliff assessment by structurally more conservative and intuitive similarity criteria. For example, a substructure-based similarity criterion has been introduced on the basis of the matched molecular pair (MMP) formalism ^12, 13^. An MMP is defined as a pair of compounds that are only distinguished by a structural change at a single site ^12^, i.e., the exchange of a substructure, which is termed a chemical transformation ^13^. Importantly, the presence of a defined substructure relationship such as the formation of an MMP can also be applied as a similarity criterion. For the definition of activity cliffs, transformation size-restricted MMPs have been introduced in which transformations are limited to relatively small and chemically meaningful replacements ^14^. Figure 1A and 1B show exemplary Tanimoto similarity- and MMP-based activity cliffs, respectively. The latter activity cliff category has been termed MMP cliff ^14^. The similarity criterion underlying MMP cliffs is simple and intuitive. MMP cliffs are often found to further improve the chemical interpretability of activity cliffs compared to cliffs defined on the basis of calculated similarity values ^4, 14^.

Another intuitive categorization of activity cliffs has been introduced on the basis of consistently defined scaffolds (obtained from compounds by removal of R-groups) ^15^ and the presence of different scaffold/R-group relationships, as illustrated in Figure 1C ^16^. This categorization makes it possible, for example, to distinguish activity cliffs that are caused by R-group replacements, small chemical changes in core structures, or chiral centers ^16^.

Moreover, activity cliffs can also be defined by comparing compound binding modes in complex ligand/target X-ray structures and calculating their 3D similarity ^17, 18^, as shown in Figure 1D. These "3D cliffs" enable the interpretation of activity cliff formation on the basis of experimentally observed ligand-target interactions and substantially add to the ligand-centric view of 2D activity cliffs. Although the Protein Data Bank ^19^ provides a steadily growing source of public domain 3D structures of therapeutically relevant proteins, only small numbers of activity cliffs can be rationalized in three dimensions and compared to 2D cliffs. In addition, 3D cliffs also rely on the calculation of similarity values. The quantification of the 3D similarity of compound binding modes is a fairly complex task because positional and conformational changes need to be taken into account.

Taken together, the examples in Figure 1 illustrate that activity cliffs can be defined in rather different -and more or less intuitive- ways, depending on the applied similarity criteria.

### Single-target activity

Based on the original definition of activity cliffs, both compounds are required to be active against a specific target ( *vide supra*). Several extensions of the activity cliff concept have been introduced that depart from this theme (for example, by considering selectivity against a pair of targets instead of single-target activity) ^3^. In principle, there is no requirement to exclusively consider active compounds for activity cliff assessment. Rather, active and inactive compounds might also be compared, provided confirmed inactive compounds are available for a given target ^20^. For SAR analysis in medicinal chemistry, the identification of small structural changes that render compounds active or inactive is of high interest, and the inclusion of confirmed inactive compounds further increases the frequency of activity cliff formation and hence our knowledge base ^20^. However, if inactive compounds are taken into consideration, a potency difference criterion is no longer applicable to define activity cliffs. Instead, a potency threshold must be set for active compounds as a cliff criterion. For example, one might require an active compound to have a potency of at least 100 nM to qualify for the formation of an activity cliff with an inactive one. The choice of this threshold is essentially subjective and it might be adjusted, depending on the application. It should also be noted that only small numbers of confirmed inactive compounds are typically available from compound optimization projects. Rather, confirmed inactive compounds mostly result from biological screening campaigns. Thus, care must be taken to obtain high-confidence activity data. For example, confirmatory bioassays from PubChem ^21^ presently provide a source of confirmed inactive compounds for more than 100 different targets.

### Isolated versus coordinated activity cliffs

**Figure 2.**
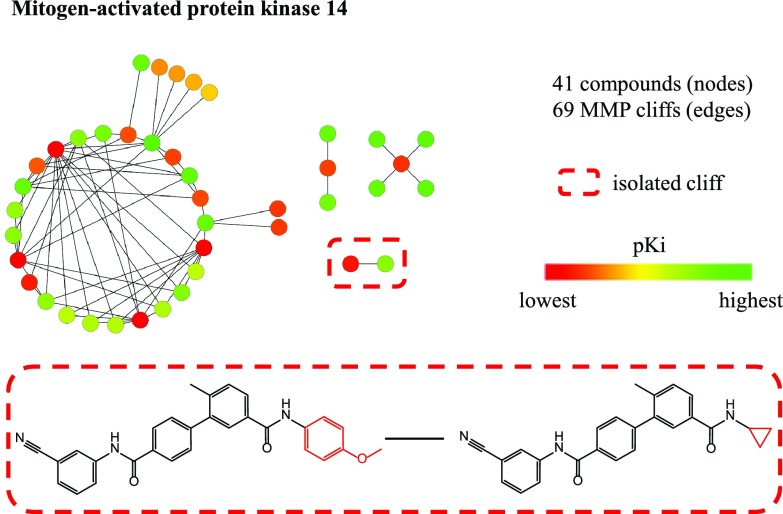
Isolated versus coordinated cliffs. MMP cliffs involving inhibitors of mitogen-activated protein kinase 14 are shown in a network representation. Nodes represent compounds that are connected by edges if they form an MMP cliff. Furthermore, nodes are color-coded according to pK _i_ values of all inhibitors applying a continuous color spectrum from red (lowest potency) via yellow (medium) to green (highest). Only one isolated activity cliff was detected in the inhibitor data set (indicated by a dashed red box). All other activity cliffs were formed in a coordinated manner involving multiple active compounds. Structures of the inhibitors forming the isolated activity cliff are shown.

The definition of activity cliffs on the basis of compound pairs might imply that cliffs are mostly formed in an "isolated" manner. This means that cliff partners are only involved in a single activity cliff and have no structural neighbors with large potency differences. However, this is clearly not the case. For example, series of highly and lowly potent structural analogs have been identified in a variety of compound data sets that form multiple and overlapping activity cliffs ^22^, giving rise to the notion of "coordinated" activity cliffs ^23^. Figure 2 shows an example of a compound set in which highly coordinated activity cliffs are formed. Higher-order activity cliff configurations involving multiple compounds are of particular interest for medicinal chemistry, given their high SAR information content. Such activity cliff arrangements can be systematically explored through data mining ^22, 23^. On the basis of our most recent survey ( *vide infra*), only small proportions of activity cliffs are formed in isolation.

## Preferred definition

As discussed above, there are many different ways to represent activity cliffs. Is it, then, possible to formulate a generally preferred detailed definition? The answer is: in principle, no; in practice, yes. From first principles, one is unable to determine (at least at present) how compound similarity should best be accounted for. In addition, setting the potency difference criterion for meaningful activity cliff and SAR analysis is subject to heuristic approximations. However, on the basis of our experience with a variety of data analyses and practical applications, we generally prefer the following activity cliff definition ^10^:

*(a) Similarity criterion: Formation of a size-restricted MMP* ^14^.

*(b) Potency difference criterion: At least two orders of magnitude.*

*(c) Activity measurements: Equilibrium constants.*

This MMP cliff definition is conservative both from a compound similarity and activity data perspective and favors chemical interpretability of activity cliffs.

## Frequency of occurrence

How often are activity cliffs found in bioactive compounds? What is the proportion of active compounds that participate in the formation of cliffs? Up-to-date results providing answers to these and other questions are reported in Table 1. These results were obtained from a large-scale analysis of compound data sets extracted from ChEMBL ( https://www.ebi.ac.uk/chembl/) ^24^. Further details are provided in the legend of Table 1. For this survey, equilibrium constants were exclusively used and a potency difference of at least two orders of magnitude was required. Thus, the MMP cliffs reported in Table 1 correspond to our preferred activity cliff definition ( *vide supra*). It should be noted that 3D cliffs are statistically underrepresented compared to 2D activity cliffs and that their frequencies of occurrence should not be directly compared. Depending on the molecular representations used, between 5.2% and 6.8% of all qualifying pairs of similar compounds form activity cliffs. The molecular representation dependence of activity cliff assessment is also reflected by the percentage of compounds that participate in activity cliffs, which ranges from 27.6% for MMP- over 35.3% for ECFP4- to 41% for MACCS-based cliffs. MMP cliffs occur with slightly lower frequency than fingerprint-based cliffs and involve a smaller proportion of active compounds. Nevertheless, on average, MMP cliffs are formed by on average every fourth active compound across many different data sets. Hence, even on the basis of this conservative assessment, *activity cliffs frequently occur and provide direct access to SAR information*. We also note that ~96.5%–98.6% of all 2D cliffs are not formed in isolation but in a coordinated manner involving more than two compounds. The example in Figure 2 illustrates that the degree of coordination is often high. For public domain 3D cliffs, the rate of isolated activity cliffs is much higher (20.4%) than for 2D cliffs. This is likely the case because structurally distinct ligands are often chosen for crystallization in order to explore different compound binding modes.

**Table 1.** Activity cliff frequency.

Types	Percentage (%)			
	Activity cliffs	Cliff-forming compounds	Isolated cliffs	Coordinated cliffs
**MACCS**	6.8	41.0	1.4	98.6
**ECFP4**	5.5	35.3	2.2	97.8
**MMP cliffs**	5.2	27.6	3.5	96.5
**3D cliffs**	8.5	13.4	20.4	79.6

The average frequency of occurrence of activity cliffs and cliff-forming compounds is reported for 129 target sets ^4^ and different molecular representations including the MACCS and ECFP4 fingerprints and MMPs. The proportion of activity cliffs was calculated on a per-target basis relative to the total number of compound pairs meeting similarity criteria and the proportion of cliff-forming compounds relative to all active compounds. Also reported is the propensity of 3D activity cliffs based on comparison of ligand binding modes in complex X-ray structures. In each case, isolated and coordinated activity cliffs are distinguished. Potency difference criterion: at least 100-fold on the basis of equilibrium constants. Similarity criteria: For MACCS and ECFP4, Tc values of at least 0.85 and 0.56, respectively ^4^; for 3D cliffs, a binding mode similarity coefficient of at least 0.80 ^17^; for MMP cliffs, formation of a transformation size-restricted MMP ^14^. Target sets: A target set is defined as a set of compounds with activity against the same target. Target sets were extracted from ChEMBL ^24^ on the basis of two selection criteria: Each set had to contain at least 100 compounds and for all compounds equilibrium constants had to be available. The 129 target sets included more than 35,000 unique compounds ^4^.

## Utilization

**Figure 3.**
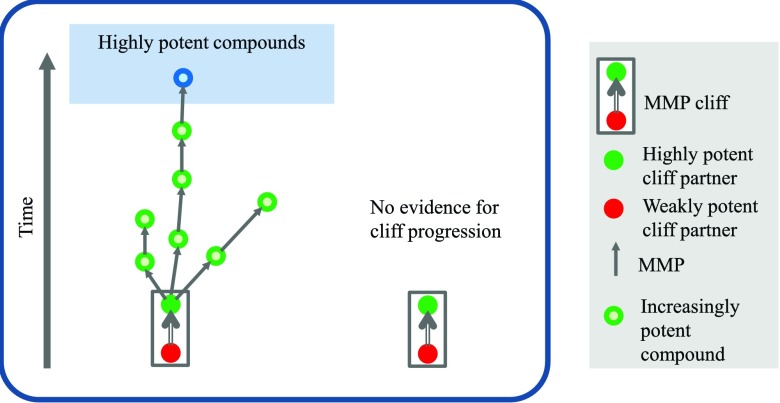
Progression and utilization of activity cliffs. Progression of activity cliffs over time is evaluated by searching for analogs of highly potent activity cliff partners. One of the compound pathways originating from the activity cliff on the left is leading over a sequence of analogs to one of the most potent data set compounds. For the activity cliff on the right, no analogs of the highly potent cliff partner are detected. Thus, in this case, there is no evidence for the potential utilization of activity cliff information.

Given the considerable frequency with which activity cliffs are formed in different compound sets, a key question is to what extent activity cliff information might currently be utilized in the practice of medicinal chemistry? This question is very difficult to answer since it is hardly possible to systematically track this information with medicinal chemists on a per-project basis. However, data mining studies can provide at least some evidence for the potential utilization of activity cliffs. In a recent study, activity cliffs were systematically identified in compound data sets evolving over time ^25^. For each highly potent activity cliff partner, it was determined whether structural analogs of this compound were reported after the activity cliff became available. If structural analogs of a highly potent cliff partner were detected in subsequent years, the possibility existed that activity cliff information provided a starting point for further compound optimization. Alternatively, if no such analogs were identified, no evidence existed for activity cliff progression, as outlined in Figure 3. On the basis of this analysis, evidence for the utilization of activity cliffs was only available for 25% of all available cliffs. By contrast, no evidence for cliff progression was detected for the remaining 75% of activity cliffs ^25^. Thus, in light of these findings, *we would conclude that existing activity cliff information is currently under-utilized in the practice of medicinal chemistry*. It is apparently difficult to bridge between data mining investigations and practical medicinal chemistry applications and consider information from the public domain early in the course of compound optimization projects. This would recommend striving for much closer links between chemoinformatics and practical medicinal chemistry.

Another related question should also be of interest. If activity cliff information is utilized, is there an "SAR evolution advantage" detectable compared to other optimization efforts not involving activity cliff compounds? To answer this question, the computational compound pathway model schematically shown in Figure 3 was applied to monitor the progression of activity cliffs towards the most potent compounds in a data set and compare activity cliff-dependent and -independent pathways ^26^. Therefore, for each active compound, a search for series of pairwise similar compounds (MMP sequences) was carried out that ultimately reached one of the 10% most potent compounds in a data set, thus delineating putative compound optimization paths in accord with the pathway model in Figure 3. Although the mean potency of activity cliff compounds and other active compounds was overall very similar, compound pathways originating from 54% of all activity cliffs successfully reached highly potent compounds, compared to only 28% of pathways originating from compounds not involved in cliff formation ^26^. *Hence, activity cliff-dependent pathways reached highly potent compounds with higher frequency than cliff-independent pathways, indicating the presence of activity cliff-associated SAR advantages*.

## Target distribution

**Figure 4.**
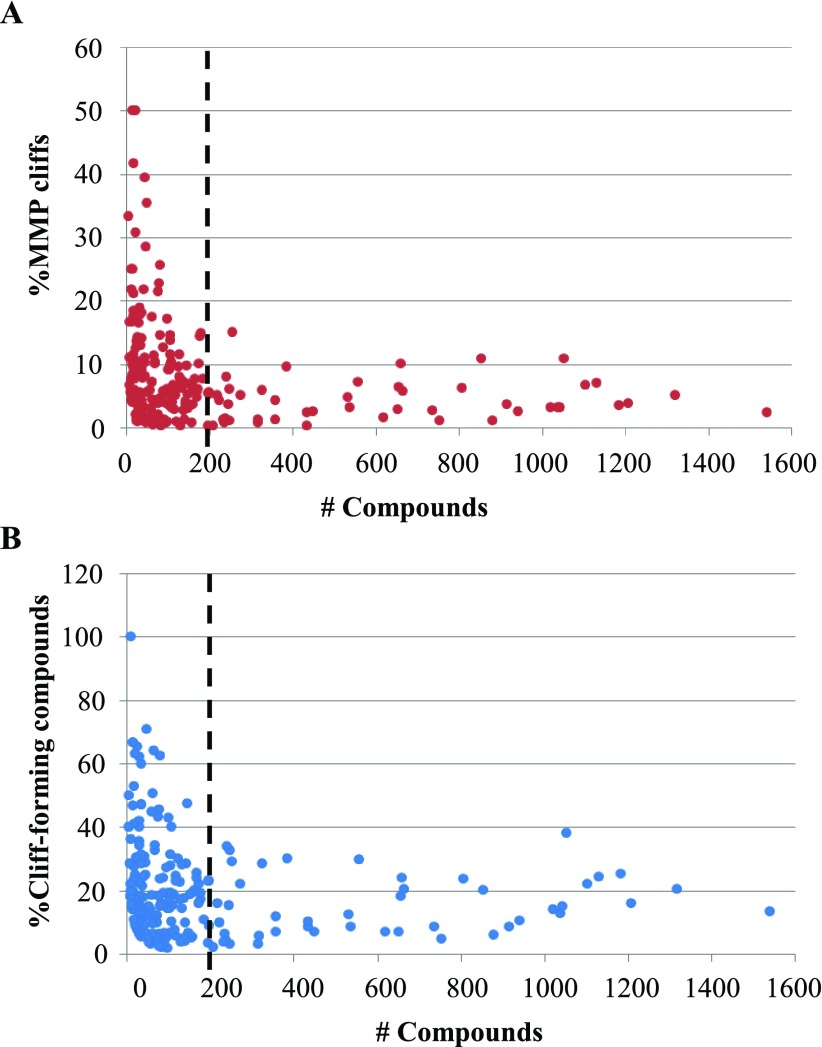
MMP cliffs in target sets of increasing size. In ( **A**) and ( **B**), the size of target sets is compared to the proportion of MMP cliffs and cliff-forming compounds they contain, respectively. Dots represent individual target sets. Dashed vertical lines mark a target set size of 200 compounds. The analysis was based on 218 target sets with available equilibrium constants extracted from ChEMBL ^24^.

**Figure 5.**
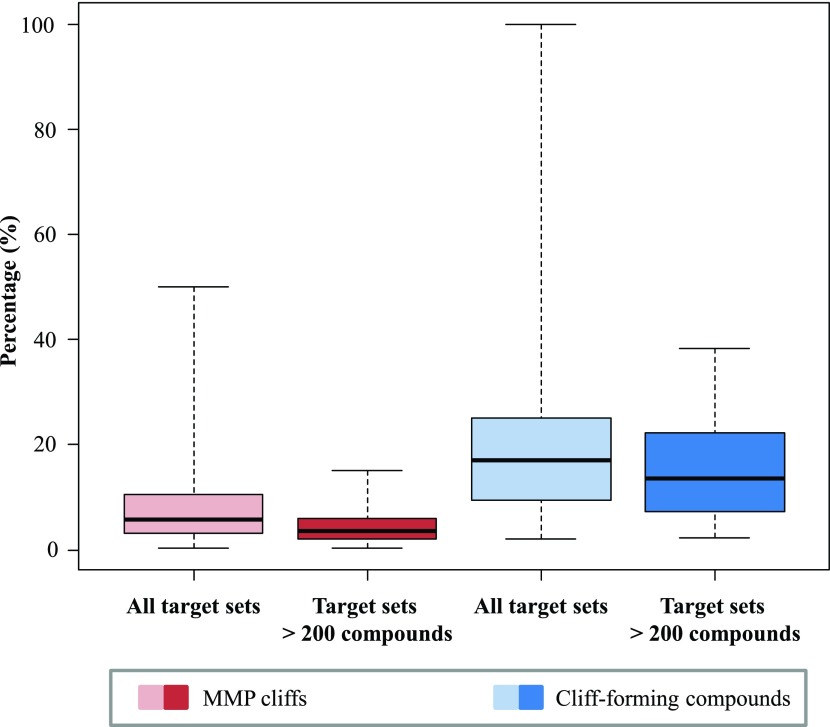
Distribution of MMP cliffs over target sets. The distributions of MMP cliffs (red) and cliff-forming compounds (blue) over target sets are reported as box plots. Each box plot provides the smallest value (bottom line), lower quartile (lower boundary of the box), median value (thick line), upper quartile (upper boundary of the box), and the largest value (top line). The dashed line indicates the value range.

Are activity cliffs differently distributed in compounds active against different targets? This is another question of considerable interest for medicinal chemistry, which has only recently been addressed ^4^. Figure 4 reports the distribution of the frequency of occurrence of MMP cliffs and, in addition, compounds participating in cliff formation for more than 200 different target sets of increasing size (each target set consists of compounds active against a specific target). The proportion of MMP cliffs relative to all MMPs and the percentage of activity cliff compounds among all active compounds were monitored. In small compound sets, significant frequency fluctuations were observed, as one would expect (for statistical reasons). By contrast, the distribution of activity cliffs and cliff-forming compounds was relatively stable for target sets containing 200 or more compounds. The box plot representations in Figure 5 indicate that *there is surprisingly little variation in the frequency of activity cliffs across many different targets for data sets of moderate to large size* (despite the presence of many different specific ligand-target interactions and binding constraints).

## Conclusions

Herein, we have focused on the activity cliff concept and provided a further refined view of activity cliffs. For medicinal chemistry, activity cliffs are of particular interest because they are associated with high SAR information content. For a meaningful assessment of activity cliffs, similarity and potency difference criteria need to be clearly defined. Care must be taken to utilize high-confidence activity data for activity cliff analysis. However, similarity assessment is the most critical step in activity cliff analysis. Although calculated similarity values can be conveniently used to describe activity cliffs, they often limit the interpretability of activity cliffs in medicinal chemistry. Therefore, substructure-based activity cliff definitions have been introduced such as MMP cliffs that further support chemical interpretation. Activity cliffs are formed with relatively high frequency among active compounds, indicating that they provide a substantial source of SAR information. In fact, if activity cliff information is utilized, as assessed on the basis of pathway modeling, compound paths originating from activity cliffs more frequently yield highly potent compounds than optimization paths originating from other active compounds. However, there currently is no evidence for utilization of about three quarters of activity cliffs in compound data sets evolving over time, which indicates that available activity cliff information is under-utilized in the practice of medicinal chemistry. Thus, there should be significant potential for further improvement of compound optimization efforts by taking activity cliff information from data mining into consideration. It has also been determined that activity cliffs are relatively evenly distributed across compounds active against a variety of targets, perhaps surprisingly so. In summary, the activity cliff concept provides an intuitive access to SAR information and can be evaluated from different perspectives. Recent analyses have yielded in part unexpected results that further differentiate our current view of activity cliffs and associated SAR features.
