# Supplementary material for: Advancing the activity cliff concept
Source: F1000Res. 2013 Sep 30;2:199. [Version 1] doi: 10.12688/f1000research.2-199.v1 (PMC3869489; doi:10.12688/f1000research.2-199.v1)
Supplement: Peer review history

# Peer review: Referee response for version 1

**Published:** 2013-10-16

**Authors:** Meir Glick

**Affiliations:** 1 Center for Proteomic Chemistry, Novartis Institutes for BioMedical Research, Cambridge, MA, USA

The commentary provides an up to date view of activity cliffs and assess their prevalence in ChEMBL. In addition, it discusses how activity cliffs can be defined using potency and similarity criteria. The paper is well written. 

My only suggestion is to make table 1 more detailed; that is to include the activity cliff frequency around target families or even around the 129 individual target sets.

I have read this submission. I believe that I have an appropriate level of expertise to confirm that it is of an acceptable scientific standard.

# Peer review: Referee response for version 1

**Published:** 2013-10-09

**Authors:** Patrick Walters

**Affiliations:** 1 Vertex Pharmaceuticals, Inc., Cambridge, MA, USA

This paper provides an overview of the concept of activity cliffs in structure-activity relationships and provides a number of examples of how the technique can be applied in a drug discovery setting. Activity cliffs can provide a means of better understanding the relationships between chemical structure and biological activity, as well as identifying regions of a molecule that are critical to activity. The review is directed toward the non-expert and should provide an adequate background for those unfamiliar with the topic. Those familiar with activity cliffs may want to consult more specialized reviews written by the same authors. This review is well-written, easy to follow, and provides many key references for those who would like to learn more about the topic.

The authors’ breakdown of the activity cliff concept into components will be helpful for those unfamiliar with the topic. It may be useful to point out that utilizing log differences in activity can make activity cliff scores more intuitive to chemists and biologists. Those with a limited knowledge of chemoinformatics will appreciate the discussion of fingerprint similarity. However, a figure might make these concepts even more clear. The authors' point out that " *calculated fingerprint Tc values are often difficult to interpret from a medicinal chemistry point of view*". In my experience, Tanimoto similarity typically makes sense, this sentence might be better as " calculated fingerprint Tc values are *sometimes* difficult to interpret". The authors mention matched molecular pairs (MMP) as a means of expressing molecular similarity.

In addition to references 12 and 13, it would be useful to include what I consider to be the seminal paper on matched molecular pairs.

Leach, A. G.; Jones, H. D.; Cosgrove, D. A.; Kenny, P. W.; Ruston,L.; MacFaul, P.; Wood, M.; Colclough, N.; Law, L. Matched Molecular Pairs as a Guide in the Optimization of Pharmaceutical Properties; A Study of Aqueous Solubility, Plasma Protein Binding and Oral Exposure. J. Med. Chem. 2006, 49, 6672–6682.

As chemists and biologists pursue more complex targets, it is necessary to increase the sophistication of our SAR analysis. I appreciate the fact that the authors point out how activity cliffs can also be used to examine multi-target activity relationships and links between sometimes-distant functional groups.

I agree with the authors' assessment that the activity cliff concept is underutilized in medicinal chemistry. Hopefully with the introduction of robust commercial software tools for the identification and exploitation of activity cliffs, the technique will be more widely used.

This review provides and excellent introduction to activity cliffs for those unfamiliar with the method. The paper is well referenced and will hopefully inspire a new group of users to continue to advance the method.

I have read this submission. I believe that I have an appropriate level of expertise to confirm that it is of an acceptable scientific standard.

# Peer review: Referee response for version 1

**Published:** 2013-10-07

**Authors:** Gerhard Müller

**Affiliations:** 1 Medicinal Chemistry, Mercachem, Nijmegen, Netherlands

Ye Hu, Dagmar Stumpfe, and Jürgen Bajorath elaborate in their commentary entitled ' ***Advancing the activity cliff concept' ***on the application of an abstract chemoinformatics-based analysis of differentiated structure-activity relationships within given compound sets. Given the high level of complexity and the expert computational chemistry knowledge often required to comprehend and to apply those latest developments in the field of chemoinformatics, I consider this commentary as extremely helpful in bridging exactly that obvious gap between the practitioners of bioactive compound data mining on the one hand, and the laboratory-based practitioners of medicinal chemistry on the other hand, respectively.

Within the context of today’s practice of medicinal chemistry, the sound and conclusive interpretation of structure-activity data is one of the most relevant, though challenging assignments in any small molecule lead finding and optimization campaign. In the course of those projects, involved teams are permanently required to make high-impact decisions on e.g. further prosecution of specific chemotypes based on systems that are, from an experimental point of view, notoriously under determined. Hence, it is of great value to account for all publicly available information on the compound-centric aspects in a given drug discovery project in order to make the most educated decision, specifically on the 'tractability' of a given chemotype.

More specifically, compound classes that after a certain chemical elaboration fail to exhibit a differentiated activity profile, i.e. do not show activity cliffs, should not be prosecuted any further since all too often those 'flat SAR' or 'rolling-hill' analogues were repeatedly identified as cul-de-sac series in many lead finding campaigns. In that context, the authors provide very helpful guidelines for the medicinal chemistry community on how to access the concept of activity cliffs by sharing expert insights they have gained over many systematic studies on practical aspects such as choosing the right potency difference and molecular similarity criteria to reliably define activity cliff patterns. Different molecular similarity metrics are introduced and comparatively ranked on their applicability and interpretability within a potential medicinal chemistry project. We all might have heard about Tanimoto, MACCS and ECFP4 fingerprints; but I assume that not too many of us are acquainted to the MMP (matched molecular pair) concept and utilize that concept pro-actively in pursued lead finding and optimization campaigns.

The group around Jürgen Bajorath actively de-mystifies those similarity concepts for us by providing quantitative measures on the inter-relation, thereby sharing their experience on the practicability of all those computational-based approaches. And for a medicinal chemist it is inspiring to see how e.g. scaffold-based cliffs unfold in a comprehensible way. I find it especially useful that the authors share their preferred definitions and settings in terms of applied similarity criteria, potency difference criteria, as well as activity measurements, thus highlighting scope and limitations of the underlying methodology. The more interested reader who wants to get really deeply involved into algorithmic details or further applications of the activity cliff concept is referred to the most recent and relevant research references.

Within the framework of a baseline study on 35000 bioactive compounds acting at 129 targets in the ChEMBL database, the overall occurrence of activity cliffs is analysed underlining the finding that activity cliffs occur quite frequently and contain valuable intrinsic SAR information that is not applied systematically. It becomes apparent that activity cliff-dependent optimization pathways seem to have a cliff-associated SAR advantage over other optimization strategies. Based on those findings, I can only support the claim that is made by the authors, notably to establish much closer links between chemoinformatics and practising medicinal chemists for a better and earlier integration of existing activity cliff information into pursued projects.

Despite the multiple and rather different ways towards activity cliffs, the concept as such becomes a tangible and intuitive asset that should be applied more frequently in the today’s pre-clinical discovery arena.

I see especially the attempt of reaching highest user-friendliness for a putative end-user that renders this commentary as very helpful to get the medicinal chemistry community alerted for this useful but still under-appreciated concept. The group around Jürgen Bajorath undoubtedly qualifies as an advocate in that sense, and the community of practising medicinal chemists should start to move in their direction accordingly.

I have read this submission. I believe that I have an appropriate level of expertise to confirm that it is of an acceptable scientific standard.
